# Co-Evaluation of Peripapillary RNFL Thickness and Retinal Thickness in Patients with Diabetic Macular Edema: RNFL Misinterpretation and Its Adjustment

**DOI:** 10.1371/journal.pone.0170341

**Published:** 2017-01-23

**Authors:** Hyun Seung Yang, Jong Eun Woo, Min-ho Kim, Dong Yoon Kim, Young Hee Yoon

**Affiliations:** 1 Department of Ophthalmology, Seoul Shinsegae Eye center, Eui Jung Bu, Gyeonggi-do, South Korea; 2 Department of Ophthalmology, University of Ulsan, College of Medicine, Ulsan University Hospital, Ulsan, South Korea; 3 Department of Statistics, Biomedical Research Center, University of Ulsan, College of Medicine, Ulsan University Hospital, Ulsan, South Korea; 4 Department of Ophthalmology, Chung-buk National University, College of Medicine, Cheong-ju, South Korea; 5 Department of Ophthalmology, University of Ulsan, College of Medicine, Asan Medical Center, Seoul, South Korea; University of Michigan, UNITED STATES

## Abstract

We investigated the relationship between the peripapillary retinal nerve fiber layer and peripapillary retinal thickness in patients with diabetic macular edema. Fifty eyes (group I) with non-proliferative diabetic retinopathy and diabetic macular edema receiving intravitreal anti-VEGF injection, and 90 eyes (group II) without diabetic macular edema were included in this case-control study. The peripapillary retinal nerve fiber layer thickness, peripapillary retinal thickness, and a new retinal nerve fiber layer index using a modeled relationship between the two parameters were evaluated with spectral-domain optical coherence tomography, at baseline and at the 6-month follow-up. In group I, the peripapillary retinal nerve fiber layer thickness decreased from 126.4 μm at baseline to 117.6 μm at 6 months (p < 0.001), while the peripapillary retinal thickness decreased from 376.0 μm at baseline to 359.6 μm at 6 months (p < 0.001) after intravitreal anti-VEGF injection. In group II, however, both the parameters remained stable at the 6-month follow-up (100.7 to 102.1 μm and 311.1 to 316.2 μm, respectively, and all p > 0.01). Analysis with the new index to adjust for retinal edema showed no significant change from baseline to 6 months in both groups (p = 0.593 and p = 0.101, respectively). The peripapillary retinal nerve fiber layer thickness is strongly affected by the peripapillary retinal thickness. Therefore, the measured changes in peripapillary retinal nerve fiber layer thickness may not represent the real gain or loss of the retinal nerve fiber layer. Therefore, the new retinal nerve fiber layer index, which corrects for the component of macula edema, could be a better means of assessing the changes of peripapillary retinal nerve fiber layer thickness in patients with diabetic macular edema.

## Introduction

Diabetic retinopathy (DR) has many elements that suggest chronic neurodegeneration as well as glaucoma and retinitis pigmentosa, including: neural apoptosis, loss of ganglion cell bodies, reduction in thickness of the inner retina, glial reactivity, neurofilament abnormality, slowing of optic nerve retrograde transport, changes in electrophysiological activity, and resultant deficits in perception [1]. Optical coherence tomography (OCT) can be used to detect gradual retinal neural tissue loss by more precise and easier measuring of RNFL thickness in DR patients with or without treatment. Dijk et al. reported that RNFL thickness decreased with DR progression [2]. Oshitari et al. showed that peripapillary RNFL thickness (pp-RNFLT) was also lower in early DR patients than in normal or non-DR patients [3]. Neurodegenerative changes in DR also may be affected by the severity and progression of DR, or the various treatments such as intravitreal injection and panretinal photocoagulation (PRP) [2,4–6]. While some studies showed that peripapillary RNFL thickness increased after PRP, other studies showed variable results after intravitreal anti-VEGF injection [7–10].

One of the reasons behind this discrepancy may be the retinal edema before and after DR treatment. As the RNFL is a part of the retina, the structural changes in the retina with or without treatment in DR patients may affect the measurements of RNFL thickness. Previous studies have suggested a possible relationship between foveal edema and pp-RNFLT, but the result was not quite satisfactory [8,10]. However, because of possible individual discrepancy between foveal edema and peripapillary edema, peripapillary retinal edema, not generalized central macular edema, is believed to have more a direct relation with the pp-RNFLT.

Thus, the current study investigates the topographic changes, including those in pp-RNFLT and retinal thickness, in DR patients with or without diabetic macular edema (DME). We also investigate a pp-RNFLT correction using the relationship between the peripapillary RNFL and topographic findings including retinal edema using spectral domain (SD) OCT.

## Patients and Methods

### Patients

This retrospective, observational, single-center case-control study reviewed the electronic clinical records of consecutive patients (>20 years of age) diagnosed with type II DM and diabetic retinopathy graded as moderate to severe non-proliferative diabetic retinopathy (NPDR) at the Asan Medical Center, Seoul, South Korea from July 2012 to September 2013. This study was performed in accordance with the 1975 Declaration of Helsinki and its 1983 revision and was approved by the Institutional Review Board of the Ulsan University Hospital in Ulsan, South Korea (UUHI 2015-03-008).

Patients with DME with > 300 μm central foveal thickness (CFT) were required to have been followed for at least 6 months including at least 2 SD-OCT follow-up examinations under the anti-VEGF treatment based on clinical conditions. Patients with moderate to severe NPDR without DME (CFT ≤ 300 μm) were included as controls. Patients were excluded from this study if they had glaucoma before and after treatment or observation, high intraocular pressure (>21 mmHg), significant epiretinal membrane around the peripapillary area, high myopia with an axial length > 26.5 mm or refractive error > -6.0 D, intraocular trauma, inflammation, previous surgery, ocular infection, choroidal atrophy, retinal detachment, or severe systemic problems including uncontrolled diabetes (HbA1c > 8.0%). Eyes that underwent pan-photocoagulation (PRP) or vitrectomy before or during follow-up were also excluded.

We reviewed 71 consecutive diabetic eyes with DME and 163 eyes without DME. Among the eyes with DME, 21 eyes were excluded [3 for vitrectomy, 1 for normal tension glaucoma (NTG), 2 for open angle glaucoma (OAG), 1 for neovascular glaucoma (NVG), 2 for increased intraocular pressure (IIOP), 2 for poor OCT images, 7 for follow-up loss and 3 for PRP), and among the eyes without DME, 73 eyes were excluded (8 for vitrectomy, 2 for NTG, 4 for OAG, 4 for NVG, 4 for IIOP, 5 for poor OCT image, 29 for follow-up loss, 11 for PRP and 6 for bevacizumab injection). As a result, 50 eyes with DME and 90 without DME that met the inclusion criteria were included in this study.

Intravitreal anti-VEGF injection was administered by a retinal specialist (YHY) with bevacizumab (0.125mg/0.05mL) by the one plus pro re nata (PRN) method. (in case of decrease in vision > 1 line or increase in CFT > 300 μm in OCT images.)

Each study patient underwent a comprehensive ophthalmological examination, including a review of medical and clinical histories, measurement of best corrected visual acuity (BCVA), slit-lamp biomicroscopy, refraction, dilated fundoscopy, intraocular pressure measurement and OCT before treatment, and at 6 months after treatment. BCVA was measured using a standard Snellen unit chart. For statistical analysis, the results were converted to the logarithm of the minimal angle of resolution (logMAR).

### OCT measurements

**Fig 1:**
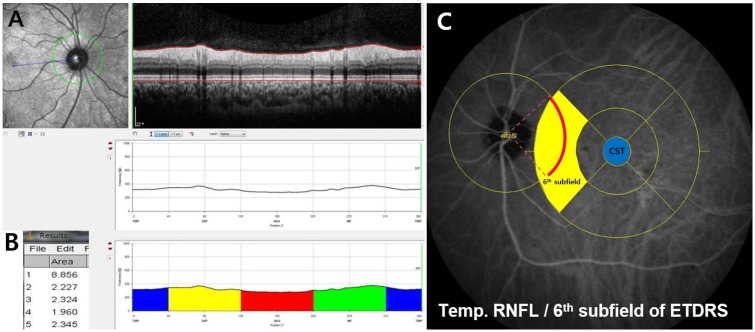
A) Peripapillary retinal thickness profile obtained automatically by changing the layer box from retinal nerve fiber layer into retina. B) The automatically calculated areas of a pixel unit in the peripapillary quadrant using the image J program (1 = total, 2 = temporal blue area, 3 = superior yellow area, 4 = nasal red area, and 5 = inferior green area). C) Overlapping portion of the temporal retinal nerve fiber layer (RNFL) thickness and the 6^th^ subfield area (yellow area) in the Early Treatment Diabetic Retinopathy Study (ETDRS). The blue area represents the central subfield thickness (CST).

At least every 6 months, SD-OCT (Spectralis, Heidelberg Engineering, Heidelberg, Germany) examinations were performed to measure central subfield thickness (CST), pp-RNFLT, and peripapillary retinal thickness (pp-RT), utilizing the AutoRescan mode with active eye tracking. Peripapillary choroidal thickness was also measured using the extended depth imaging method. The OCT images were generated using the horizontal SD-OCT cross section (15 lines spaced 250 microns apart) for macular thickness. For better image quality, 25–30 frames were averaged for each B-scan. Quality criteria included an automatic real-time score of 16, and signal to noise ratio of 15 dB or higher. Pp-RNFLT was measured in 4 sectors: temporal, superior, nasal and inferior. Sixteen consecutive circular B-scans centered at the optic disc were automatically averaged (3.4 mm diameter, 768 A-scans) after the reference lines were adjusted by a trained technician who was masked to the clinical information. The pp-RT measurements in pixel units were conducted using Image J (http://imagej.nih.gov/ij/index.html) by automatic area calculation in 4 sectors (Fig 1A and 1B) after exporting the raw data of pp-RT contour figure from the Heidelberg Eye Explorer software (ver. 1.7.1.0; Heidelberg Engineering, Heidelberg, Germany) and HRA/Spectralis Viewing Module (ver. 5.6.4.0). The area of each sector was divided by the arc-length (3.4mm×3.14 / 4) of that portion of the circle to get the average pp-RT in each sector, and the total area was divided by the circumference of the circle (3.4mm×3.14) for the average pp-RT. A pixel unit was converted into the standard unit (microns) by dividing the average thickness (pixels) in image J by the average thickness (microns) in the RNFL map. Segmentation error was observed in 12 DME eyes with thick focal posterior shadow, and 3 non-DME eyes with peripapillary retinal edema. In cases of RNFL or RPE posterior border discontinuation due to posterior shadowing of macular edema, the artificial continuous line that connected with the faint posterior border of the RPE was used by moving the posterior segment line. Fig 1C shows the relationship between the Early Treatment Diabetic Retinopathy Study (ETDRS) subfield areas and the pp-RNFLT circle. The 6^th^ subfield area (the most nasal area) in the ETDRS overlapped almost entirely with the temporal quadrant of the pp-RNFLT map.

The peripapillary choroidal thickness (between Bruch’s membrane and the inner portion of the sclera) was manually measured in 4 sectors by moving the two reference lines following the previously reported methods [11–14] using the Heidelberg’s Eye Explorer software by two authors (HSY and JEW), who were masked to the clinical information. The means of the two observer values were used in the analysis. The average choroidal thickness in the 4 sectors was calculated automatically by the Viewing Module program.

### New index of pp-RNFLT

**Fig 2:**
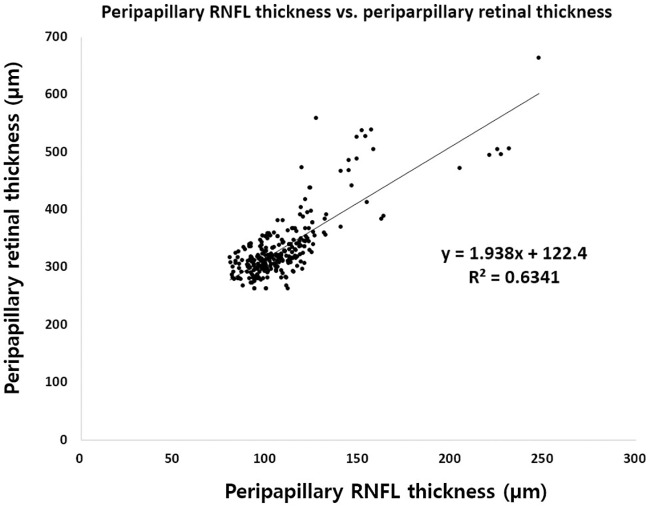
Scatter plots of generalized equations between overall peripapillary retinal thickness and retinal nerve fiber layer thickness.

A new index was calculated by dividing the pp-RNFLT of each sector by the corresponding pp-RT, using a formula derived from the generalized linear regression equations (S1 File). The changes in the average of the index of each sector were compared at baseline, and at 6 months. The data can be corrected by dividing by the inverse form of the generalized equations (Fig 2), thereby minimizing the effect of pp-RT.

$$Index\;\left(average\right)=\frac{1.94\;*\text{ Pp}-\text{RNFLT}}{\text{Pp}-\text{RT}-122.40}\;*\;100$$

We corrected each pp-RNFLT measurement using the RNFL Index (average) formula and investigated the trend of real RNFL thickness change during the 6-months follow up.

### Statistical analyses

Statistical Product and Service Solution (SPSS; v21, IBM Corp. Armonk, NY, USA) was used for the statistical analysis and if the p value was less than 0.01, it was considered to be statistically significant. Baseline characteristics were compared between groups by an independent T-test or Chi-square test. A paired T-test was used to compare the pp-RNFLT and pp-RT between the different time points: at baseline and at 6 months. A Pearson’s correlation coefficient was calculated to evaluate the relation between the RNFL and retinal thickness at the peripapillary or foveal region.

## Results

### Baseline characteristics

The average age of the subjects was 67.7 ± 12.9 years and the average follow up duration at baseline for DM patients was 14.0 ± 6.8 years. Seventy-five patients were men (53.5%) and 65 were women (46.5%) At baseline, the average HgA1c was 6.8 ± 1.2% and IOP was 15.8 ± 2.3 mmHg. The initial average mean deviation of VF was -1.93 ± 1.29 in 68 patients (48.6%). The average CST, pp-RNFLT, and peripapillary choroidal thickness at baseline were 343.2 ± 96.1 μm, 109.9 ± 23.2 μm, and 176.8 ± 21.1 μm, respectively. Table 1 indicates the baseline clinical and topographical characteristics of each group. The age, gender, initial BCVA, DM duration, HgA1c at baseline, IOP, and average choroidal thickness were not different between the groups. However, the average CST and peripapillary average RNFL thickness of group 1 were significantly thicker than those of group II (p < 0.001). The average number of intravitreal anti-VEGF injections was 2.7 ± 1.7 in 6 months in group I.

**Table 1:** Comparison of baseline characteristics between eyes with non-proliferative diabetic retinopathy with or without diabetic macular edema.

Characteristic	Group I (DME)	Group II (No DME)	p-value
Number of eyes	50	90	
Age (year)	68.2 ± 13.1	67.4 ± 13.0	.827^a^
Gender (Men/Women)	28/22	47/43	.594^b^
Initial BCVA (logMAR)	0.31 ± 0.22	0.12 ± 0.09	.007^a^
Spherical equivalent refractive error (diopters)	-1.86 ± 1.70	-1.79 ± 1.55	.284^a^
Duration of DM at baseline (years)	13.2 ± 6.6	14.4 ± 6.9	.756^a^
HbA1c at baseline (%)	6.9 ± 1.4	6.7 ± 1.1	.601^a^
IOP (mmHg)	15.6 ± 2.3	15.9 ± 2.5	.677^a^
Central subfield thickness (μm)	452.7 ± 183.2	282.5 ± 48.2	.000^a^
Average RNFL thickness (μm)	126.4 ± 34.7	100.7 ± 17.0	.000^a^
Average choroidal thickness (μm)	180.5 ± 17.5	174.8 ± 23.2	.152^a^

^a^Independent T-test; group I vs. group II;

^b^Chi-square test; group I vs. group II

DM = Diabetes Mellitus; DME = diabetic macular edema; IOP = intraocular pressure; MD = mean deviation; RNFL = retinal nerve fiber layer; VF = visual field

### Quantitative change in pp-RNFLT and pp-RT

Table 2 shows the peripapillary RNFL thickness changes during the follow up period in the 2 groups. In group I DME eyes, the average pp-RNFLT significantly decreased from baseline to 6 months (p < 0.001). When divided into 4 quadrants, only the temporal & inferior RNFL thickness also showed a similar trend (all p < 0.001) at 6 months. In comparison, in group II non-DME eyes, the average and individual quadrant pp-RNFLT showed no significant change over the follow up period (all p > 0.01). Table 3 shows the pp-RT during the follow up period in the 2 groups. While CST, pp-RT, and pp-RNFLT decreased similarly in group I DME eyes, none of those changed significantly in group II non-DME eyes.

**Table 2:** Comparison of changes in retinal nerve fiber layer thickness during 6-month follow up period in eyes with non-proliferative diabetic retinopathy with or without diabetic macular edema.

Characteristic	Group I (DME)		Group II (No DME)	
	Baseline (A)	At 6 M. (B)	Baseline (A)	At 6 M. (B)
Total RNFL thickness (μm)	126.4 ± 34.7	117.6 ± 28.8	100.7 ± 10.0	102.1 ± 12.1
P values^a^	P_A-B_ < 0.001		P_A-B_ = 0.079	
Temporal RNFL thickness (μm)	117.1 ± 43.3	101.6 ± 40.8	81.2 ± 13.8	83.0 ± 15.5
P values^a^	P_A-B_ < 0.001		P_A-B_ = 0.036	
Superior RNFL thickness (μm)	150.0 ± 53.4	140.5 ± 34.2	122.8 ± 19.3	124.2 ± 20.2
P values^a^	P_A-B_ = 0.068		P_A-B_ = 0.172	
Nasal RNFL thickness (μm)	87.6 ± 22.1	84.2 ± 23.7	70.8 ± 11.2	71.9 ± 18.9
P values^a^	P_A-B_ = 0.117		P_A-B_ = 0.426	
Inferior RNFL thickness (μm)	150.8 ± 35.9	144.2 ± 33.9	127.9 ± 17.6	129.2 ± 18.6
P values^a^	P_A-B_ = < 0.001		P_A-B_ = 0.159	

^a^Paired T-test;

DME = diabetic macular edema; M. = months; RNFL = retinal nerve fiber layer

**Table 3:** Comparison of changes in central subfield thickness and peripapillary retinal thickness during 6-month follow up period in eyes with non-proliferative diabetic retinopathy with or without diabetic macular edema.

Characteristic	Group I (DME)		Group II (No DME)	
	Baseline (A)	At 6 M. (B)	Baseline (A)	At 6 M. (B)
**Central subfield thickness (μm)**	452.7 ± 183.8	381.2 ± 100.8	285.5 ± 48.2	299.9 ± 87.8
P values^a^	P_A-B_ < 0.001		P_A-B_ = 0.038	
**Average peripapillary retinal thickness (μm)**	376.0 ± 84.4	359.6 ± 69.7	311.1 ± 24.1	316.2 ± 25.7
P values^a^	P_A-B_ < 0.001		P_A-B_ = 0.045	
Temporal (μm)	409.3 ± 75.7	377.2 ± 72.0	304.7 ± 23.9	311.4 ± 24.1
P values^a^	P_A-B_ < 0.001		P_A-B_ = 0.010	
Superior (μm)	386.7 ± 68.8	370.6 ± 45.1	331.9 ± 24.1	335.5 ± 22.3
P values^a^	P_A-B_ = 0.006		P_A-B_ = 0.109	
Nasal (μm)	329.2 ± 71.5	319.4 ± 65.0	269.7 ± 19.3	277.4 ± 31.5
P values^a^	P_A-B_ = 0.024		P_A-B_ = 0.038	
Inferior (μm)	378.6 ± 85.1	371.1 ± 63.6	338.0 ± 21.3	340.7 ± 19.5
P values^a^	P_A-B_ = 0.048		P_A-B_ = 0.219	

^a^Paired T-test, P values less than 0.01were considered statistically significant; DME = diabetic macular edema; M. = months; RNFL = retinal nerve fiber layer

### Relationship between pp-RNFLT and pp-RT, 6^th^ subfield thickness, and CST

**Fig 3:**
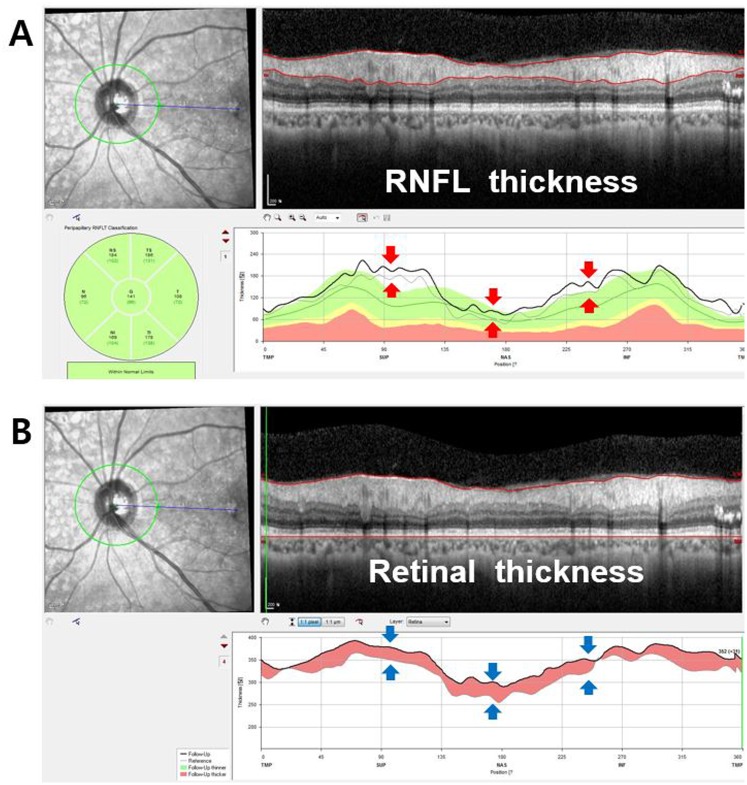
The similar profiles of RNFL thickness and retinal thickness change in the peripapillary area. A) The red arrows indicate the increase of peripapillary RNFL thickness during the 6-month follow up period (gray line at baseline and thick black line at 6 months). B) The blue arrows indicate the increase of peripapillary retinal thickness during the follow up period (gray line at baseline and thick black line at 6 months).

**Fig 4:**
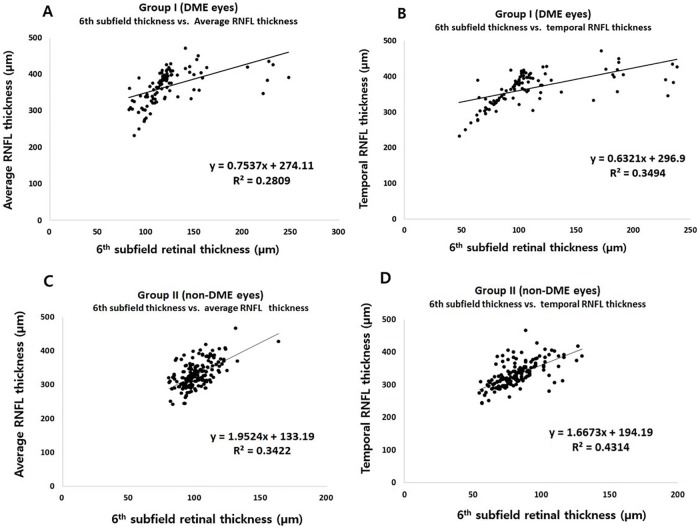
Scatter plots of generalized equations between the 6^th^ EDTRS subfield retinal thickness and peripapillary retinal nerve fiber layer thickness. A) and B) show the relationship between the 6^th^ subfield retinal thickness and average or temporal RNFL thickness in patients with diabetic macular edema (group I). C) and D) show the relationship between the 6^th^ subfield retinal thickness and average or temporal RNFL thickness in patients without diabetic macular edema (group II).

As seen in Fig 3, the RNFL thickness profile in peripapillary area closely corresponds to the peripapillary retinal thickness profile. In group I DME eyes, pp-RNFLT and pp-RT decreased significantly from baseline to 6 months (p < 0.001 and p < 0.001, respectively). However, as expected, both pp-RFNLT and pp-RT did not change in group II non-DME eyes (Tables 2 and 3). While the average pp-RNFLT showed a positive correlation with the CST (R = 0.512, p < 0.001), the temporal pp-RNFLT had a stronger correlation with CST (R = 0.558, P < 0.001) (Table 4). In both groups, temporal pp-RNFLT had a higher correlation with 6^th^ subfield thickness (R = 0.591 in group I and 0.657 in group II) than average RNFL thickness (R = 0.530 in group I and 0.585 in group II) (Fig 4).

**Table 4:** Correlation coefficient between the peripapillary RNFL thickness and topographic findings at baseline and 6 months in eyes with non-proliferative diabetic retinopathy with or without diabetic macular edema.

Factor	At baseline			At 6 month		
	Peripapillary retinal thickness (μm)	ETDRS 6^th^Subfield Retinal thickness	CST (μm)	Peripapillary retinal thickness (μm)	ETDRS 6^th^Subfield Retinal thickness	CST (μm)
Peripapillary RNFL thickness (μm)	R = 0.823 (P < 0.001)	R = 0.592 (P < 0.001)	R = 0.512 (P < 0.001)	R = 0.756 (P < 0.001)	R = 0.590 (P < 0.001)	R = 0.401 (P < 0.001)

CST = central subfield thickness; ETDRS = Early Treatment Diabetic Retinopathy Study; RNFL = retinal nerve fiber layer

### New RNFLT index for adjusting the effect of retinal edema

**Fig 5:**
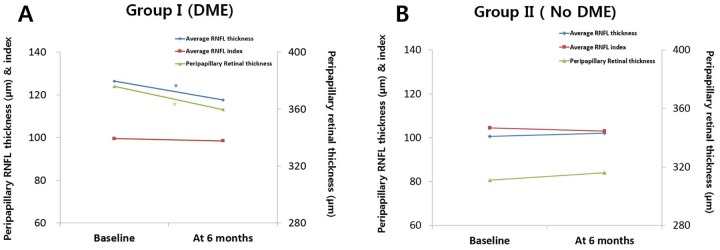
Average peripapillary retinal nerve fiber layer (RNFL) thickness, corresponding peripapillary retinal thickness, and RNFL index change during the 6-month follow up period in the 2 groups. A) Eyes with diabetic macular edema (DME) show RNFL thinning (diamond marks) after anti-VEGF injection, but RNFL index after peripapillary retinal edema correction shows no change (rectangular marks). B) Eyes with no DME show no significant change in RNFL thickness (diamond marks) relative to the retinal thickness and RNFL index (rectangular marks) shows no change, similar to that in the DME group. *P < 0.01 (P values less than 0.01 were considered statistically significant).

The new RNFLT index was calculated by using the relationship between the pp-RNFLT and the corresponding pp-RT in each patient using specific linear regression (Fig 2). In group I DME eyes, while average RNFLT decreased from baseline to 6 months, the average RNFLT Index did not show any significant decrease from baseline to 6 months (p = 0.593). In contrast, in group II non-DME eyes, the RNFLT Index remained stable throughout the 6 months (p = 0.101). Our data suggests that RNFLT could be erroneously measured when the retina is thickened by diabetic macular edema. Therefore, in such cases, the pp-RNFLT should be evaluated after adjusting for macular edema (Table 5 and Fig 5).

**Table 5:** Comparison of average changes of adjusted retinal nerve fiber layer thickness Index during 6-month follow up period in eyes with non-proliferative diabetic retinopathy with or without diabetic macular edema.

Characteristic	Group I (DME)		Group II (No DME)	
	Baseline (A)	At 6 M. (B)	Baseline (A)	At 6 M. (B)
Number of eyes	50	50	90	90
Average of Total RNFL thickness Index	99.5 ± 15.0	98.4 ± 14.8	104.5 ± 13.6	103.0 ± 13.4
P values^a^	P_A-B_ = 0.593		P_A-B_ = 0.101	
Average of Temporal RNFL thickness Index	89.9 ± 14.8	82.6 ± 16.8	84.2 ± 16.6	83.7 ± 16.5
P values^a^	P_A-B_ < 0.001		P_A-B_ = 0.460	
Average of Superior RNFL thickness Index	117.8 ± 23.5	118.4 ± 22.8	127.4.0 ± 22.5	125.6 ± 22.7
P values^a^	P_A-B_ = 0.787		P_A-B_ = 0.170	
Average of Nasal RNFL thickness Index	69.4 ± 12.5	70.7 ± 15.7	73.5 ± 13.4	72.5 ± 17.2
P values^a^	P_A-B_ = 0.626		P_A-B_ = 0.404	
Average of Inferior RNFL thickness Index	120.9 ± 25.9	122.2 ± 25.1	132.6 ± 21.3	130.4 ± 21.0
P values^a^	P_A-B_ = 0.612		P_A-B_ = 0.051	

^a^Paired T-test, p value is less than 0.01, it was considered to be statistically significant;

DME = diabetic macular edema; M. = months; RNFL = retinal nerve fiber layer

## Discussion

Recently, it has been found that retinal function loss in diabetic patients and/or those with early DR is not only due to changes in micro-vascular pathology, but also neurodegenerative change [1,6,15,16]. The degeneration of retinal ganglion cells (RGCs) has been reported to begin early after the onset of diabetes in rats, and it is suggested that since this neurodegeneration may proceed to retinal micro-vasculopathy, it may contribute to capillary degeneration [17]. Several studies also showed that RGC loss and/or decreased Rarebit visual field test results have a relation with the presence or duration of diabetic retinopathy with minimal vascular change [1,15].

The recent development of OCT has contributed to an increasing number of quantitative studies utilizing segmental analysis of each retinal layer, including the pp-RNFLT. Thus, quantitative RNFL thickness analysis has been used as the main indicator in the diagnosis and progression of glaucoma and various optic nerve diseases. In addition, there were some trials that showed that neurodegenerative changes in DR may be detected using SD-OCT in an animal model [18]. Dijk et al. and Oshitari et al. also showed that RNFL and RGC layer thickness is reduced over time in DR patients and the degree of thinning was related to the severity of DR [2,3,15].

**Fig 6:**
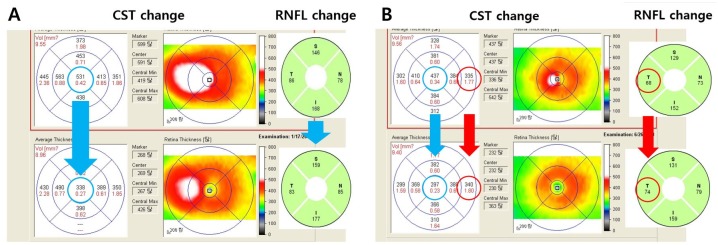
Spectral-Domain optical coherence tomography images (upper) before and (lower) 6 months after anti-VEGF injection for comparing changes between central subfield thickness (CST), temporal peripapillary retinal nerve fiber layer (RNFL) thickness in two patients with diabetic macular edema. A) Case 1: The RNFL thicknesses in all quadrants decreased following the CST decrease. B) Case 2: Even though the central subfield thickness reduced (437 μm → 297 μm) after anti-VEGF injection, the temporal RNFL thickness increased (68 μm → 74 μm), as did the 6^th^ subfield thickness (335 μm → 340 um).

However, the RNFL thickness measurement might be affected by various conditions in diabetic patients. According to a recent study, the RNFL thickness change after anti-VEGF and PRP varied. Seth et al. found that there was no significant change in the vertical cup and disc ratio of optic nerves in patients receiving multiple intravitreal anti-VEGF injections [19]. In addition, multiple intravitreal injections in patients with wet age-related macular degeneration did not lead to significant loss of the RNFL. Kim et al. and Lee et al. showed thickening of the RNFL after PRP in the average peripapillary area at the 6-month follow up and thinning of RNFL at the 1-year follow up [7,8]. Furthermore, DR patients without treatment showed a decrease in RNFL thickness due to age-related changes, neurodegeneration or retinal ischemia [1,3,9,15,20,21]. Thus, using the new automatic measurement of pp-RT, our present study directly proved that the main reason in fluctuation of the RNFL thickness measurement is the peripapillary retinal edema, which is affected by diabetic macula edema (Fig 3). Furthermore, we re-analyzed the RNFL changes in the patients who may have retinal edema by adjusting for RNFL edema component using the RNFL thickness/ retinal thickness ratio in each peripapillary quadrant sector. The results using this new index were surprisingly quite different from the non-corrected results. The pp-RNFLT changes followed the fluctuation of macular edema more precisely than peripapillary retinal edema. Fig 6A shows a classic patient whose pp-RNFLT appeared to be decreased in all 4 quadrants following the macular edema improvement after Anti-VEGF injection. However, Fig 6B shows there can be some differences between the CST and pp-RT, and the RNFL thickness has a tendency to follow the change of the pp-RT rather than that of the CST.

Thus, discrepancy between real RNFL loss and thicker RNFL change in DME might be understood using this method. We can also assume the RNFL index is the minimum changeable value correcting pp-RNFLT when we analyze the RNFL thickness change. This value is closer to the real RNFL loss or gain than measured RNFL thickness with macular edema during the follow up especially in DR patients with both of macular edema and neurodegeneration. In addition, best purpose of index is observing the general trends of real RNFL thickness change in DME or atrophic retina as well, so there also needs more specific formula to correct depending on the specific groups, periods and areas. And this study may be the first step for the correcting phenomenal RNFL thickness. Recently, Hwang el al. also showed that pp-RNFLT decreases after intravitreal injection in short term follow up which may arise from the decrease of retinal and corresponding RNFL edema [10]. However, macular edema measured as CFT and/or CST is not correlated with the peripapillary retinal edema (as in Fig 6B) so the RNFL thickness may-not be corrected using the macular thickness in their study. Furthermore, the majority of the temporal RNFL thickness is located the in 6^th^ EDTRS subfield retinal thickness (Fig 1C), i.e., there is a good relationship between the temporal RNFL thickness and corresponding pp-RT (6^th^ subfield) in Fig 4B and 4D. Regarding this, Kim et al. also suggested that the increase of RNFL after PRP may be due to the central macular edema [8]. However, they also failed to find a significant relationship between the peripapillary RNFL and central foveal thickness (R≤0.47). In addition, Hsu et al. reported that superior/inferior and temporal/nasal ratios were more reliable in evaluating peripapillary RNFL in patients with diabetic retinopathy to eliminate the effect of macular edema [22]. However, their method cannot be used to analyze each individual peripapillary quadrant and the same amount of RNFL edematous change cannot be guaranteed between quadrants. In addition, the repeatability of RNFL thickness in DME using the low-resolution model might be questionable. Regarding this, we already reported that the repeatability of RNFL and retinal thickness in DME was quite reliable using high resolution OCT in a previous study [23]. In the present study, using the relation between the pp-RNFLT and pp-RT described above we have devised a means of minimizing the effect of corresponding retinal edema and deriving the actual RNFL thickness change in DR patients with macular edema.

One of the strengths of this study was the inclusion of patients who were carefully selected according to strict criteria by two retinal subspecialists (HSY and YHY) in our Diabetic Retinopathy Clinic. In addition, the present study only focused on treatment-naïve NPDR patients, and all analyzed groups were relatively well-balanced. Moreover, the number of patients was sufficient for the analysis of the relationship between the topographic features. Despite these strengths, the present study also had some limitations, including its retrospective design. In addition, selection bias might have arisen due to the relatively high percentage of initial patients (29.6% in group I and 44.8% of group II) who were excluded from the study in order to eliminate scans with potential artifacts. In addition, the ratio of edema can be different depending on the specific layer of the retina, including the RNFL, in every quadrant, so that the equation cannot be generalized for different groups and retinal diseases. Further study regarding the segmented layer and the appropriate adjustments using this equation for the RNFL thickness in each independent quadrant is needed.

To the best of our knowledge, there have been no studies for 1) measuring the pp-RT automatically using the peripapillary retinal profile, 2) evaluating the direct relationship between the pp-RNFLT and corresponding pp-RT, and 3) correcting for the retinal edema component to more accurately evaluate the RNFL thickness change in DME patients. Thus, this study may help us in better understanding the pp-RNFLT change in the present era with early diagnosis and treatment of DME, concomitant anti-VEGF injection, and frequent comorbidity of glaucoma.

In conclusion, the pp-RNFLT is strongly affected by retina edema, especially in the peripapillary area, so that pp-RNFLT itself does not indicate the actual RNFL gain or loss in DR patients. Thus, our method for correction of pp-RNFLT using the relationship with RNFL thickness and pp-RT should be considered when performing objective analyses of the real RNFL thickness change in DR patients.

## Supporting Information

S1 File Derivation of the correction formula. (DOCX)
