# Supplementary material for: Co-Evaluation of Peripapillary RNFL Thickness and Retinal Thickness in Patients with Diabetic Macular Edema: RNFL Misinterpretation and Its Adjustment
Source: PLoS One. 2017 Jan 23;12(1):e0170341. doi: 10.1371/journal.pone.0170341 (PMC5256947; doi:10.1371/journal.pone.0170341)
Supplement: S1 File — (DOCX) [file pone.0170341.s001.docx]

**S1 file**

**Derivation of the correction formula**

The index value represents the difference between the real RNFL thickness and the calculated ideal (average) RNFL thickness. The calculated ideal value is derived from the (x, y) values of the trend line equation (fig 2).

In other words,

$$Index \left( corrected \right)= \frac{real RNFL thickness}{calculated ideal RNFL thickness} \times100$$

However, to meet the specific retinal thickness criteria (specific ideal retinal thickness = specific real retinal thickness), the retinal thickness should be 1.94 * ideal RNFL thickness + 122.40, according to the trend line. This means that the specific index formula can be expressed as

$$Index= \frac{real RNFL thickness}{\frac{real retinal thickness-122.40}{1.94}} \times100$$

After rearranging the terms, we have the formula

$$Index \left( average \right)=\frac{1.94 \times RNFL thickness}{Retinal thickness-122.40}\times100$$
